# Integrating health promotion into the educational mission: a 25-year bottom-up institutional trajectory in a Chilean university

**DOI:** 10.1093/heapro/daag039

**Published:** 2026-04-27

**Authors:** Mónica Castillo-Rosales, Angelo Araya-Piñones, Nicolas van Niekerk Bakit, Irma Brito, Marisa Afonso de Andrade Brunherotti, Verónica Alfaro-Navarro, Trinidad Bakit

**Affiliations:** Centro de Salud Estudiantil, Departamento Estudiantil, Universidad Católica del Norte, Larrondo 1281, Coquimbo 1780000, Chile; Departamento de Psicología, Facultad de Humanidades, Universidad de La Serena, Av Cisternas 1200, La Serena 1700000, Chile; Faculty of Health Sciences, Department of Kinesiology, Universidad de Atacama, Av Copayapu 2862, Copiapó 1530000, Chile; Health Sciences Research Unit: Nursing (UICISA: E), Av Bissaya Barreto 52, 3030-000 Coimbra, Portugal; Nursing School, University of Coimbra (ESEUC), Av Bissaya Barreto 52, 3030-000 Coimbra, Portugal; Department of Health Promotion, Universidade de Franca, Av Doutor Armando Sales Oliveira 201, 14404-600 Franca, São Paulo, Brazil; Departamento Estudiantil, Universidad Católica del Norte, Larrondo 1281, Coquimbo 1780000, Chile; Escuela de Obstetricia y Puericultura, Universidad de Valparaíso, Camino La Troya S/N, San Felipe 2170000, Chile

**Keywords:** health policy, health promotion, institutionalization, social determinants of health, health behaviour, health policy, planning and management

## Abstract

Although universities worldwide are increasingly adopting health promotion strategies, there remains limited longitudinal evidence on how these initiatives are institutionally integrated and sustained over time, especially in Latin American contexts. This study explores the 24-year trajectory (1999–2023) of the first Health Promotion Policy implemented at a Chilean university, providing rare insights into a long-term bottom-up institutional process. In analytical terms, the VIVE UCN Evaluation Model was applied using the milestones (M), actors (A), processes (P), and challenges (C) (MAPC) framework, a convergent mixed-methods design that combined a qualitative documentary analysis of 47 official and unofficial sources with quantitative analysis using descriptive statistics. The MAPC framework was applied to documents from 1999 to 2023, including policy texts, management reports, and evaluation records, to systematically identify milestones, actors, processes, and challenges shaping the policy trajectory. Findings reveal that community participation and collective ownership were key to the policy’s institutionalization, shifting health promotion from isolated initiatives into an educational and organizational commitment. By documenting a sustained, bottom-up experience from the Global South, this study contributes new longitudinal evidence to the field of health-promoting universities. Ultimately, integrating the health promotion policy into the educational mission demonstrates a long-term commitment to creating healthy settings and empowering the academic community to address the social determinants of health—both goals recommended by the World Health Organization for higher education institutions.

## Graphical Abstract

**Graphical Abstract:**
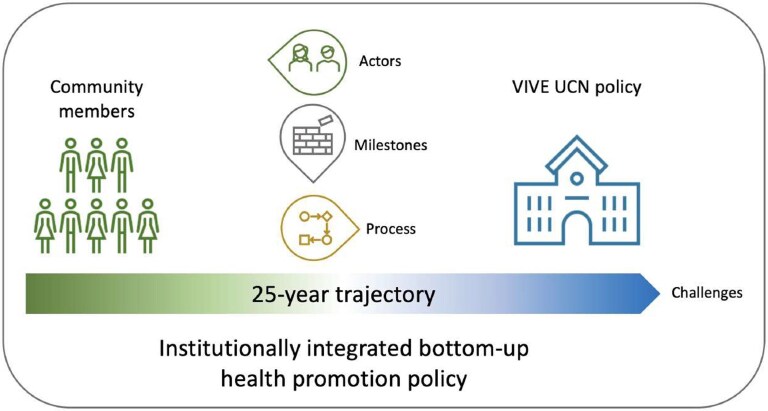

Contribution to Health Promotion

- This study traces the 25-year trajectory of a bottom-up health promotion policy institutionally integrated into a Chilean university’s educational mission.
- The findings highlight processes of appropriation, participation, and sustainability consistent with the principles of the Ottawa Charter.
- The institutionalization of health promotion illustrates the contribution of a settings-based approach to advancing health and well-being in higher education.
- This longitudinal case provides rare Latin American evidence of sustained institutional appropriation in health promotion.
- The insights from this case may inform and strengthen the development of national health promotion policies.

## Introduction

People’s health encompasses a range of factors, including social and environmental factors. Consequently, the health promotion approach has emerged as a viable strategy to strengthen social determinants (WHO 1986, McKenzie *et al*. 2023). This approach has been the focus of ongoing efforts to enhance this strategy (WHO 1997, 2013), concentrating on helping organizations and communities gain control over the factors that define health (Van Den Broucke 2020). In this context, the Pan American Health Organization (PAHO) has embraced the Sustainable Development Goals (SDGs) by renewing its health promotion strategy, which addresses the conditions in which people live, work, or study. This strategy encourages a transition from a framework centred on individual behaviour to one that prioritizes environmental and community aspects (PAHO 2022). The goal is to empower individuals and communities to take charge of their health and live fully by addressing the social determinants of health in their settings (WHO 2021).

### Health promotion among higher education institutions

The efforts of the World Health Organization (WHO) and PAHO, together with collaborations with public and private entities, have addressed the determinants of health (WHO-PAHO 2015), implemented healthy and sustainable setting-based approaches (Kokko and Baybutt 2022), and helped realize healthy lifestyles (Núñez-Rocha *et al*. 2020). In this context, health promotion aims to develop strategies that address the conditions that influence people’s development. Thus, the Ottawa Charter (WHO 1986) for Health Promotion represents a pivotal milestone in advancing the development of healthy settings. Consequently, higher education institutions (HEIs) are encouraged to collaborate with governments and local stakeholders to develop policies that address the social determinants of health. (UNESCO 2023) and to train capable, socially empowered individuals to implement innovative approaches (Okanagan Charter 2015). In this way, HEIs have progressed along the path established in the 1980s, building on PAHO’s momentum (Brito *et al*. 2022).

Within this evolving global framework, the First International Conference on Health Promoting Universities was held in England in 1996, followed by a conference in Canada, where the Edmonton Charter called on institutions to implement policies that foster healthy learning, working, and living environments (Edmonton Charter 2005). Subsequently, the 2015 Okanagan Charter urged universities to integrate health into campus culture, including administrative, operational, and academic goals (Okanagan Charter 2015). More recently, in 2025, the Limerick Framework in Ireland emphasized the need to integrate health, well-being, and sustainability considerations into all policies (Limerick Framework 2025). In Ibero-America, the genesis of the Health Promoting Universities movement dates to 2003, with the First International Congress held in Chile. Since then, 10 congresses have taken place, including the 2025 meeting in Brazil, which called for strengthening HEIs’ commitment to creating healthy settings and promoting the well-being of the university community. In scholarship on health-promoting universities, seminal work advanced a comprehensive model integrating health into university governance, curriculum, organizational culture, and community engagement (Dooris 2001a, Dooris *et al*. 2020). In parallel, Tsouros *et al*. (1998) established the strategic foundations of the movement by positioning universities as priority settings for health promotion.

In this context, HEIs are a suitable venue for implementing health promotion programmes (Taylor *et al*. 2019) due to the ease of defining action spaces (e.g. physical and academic) and of having a captive population for a specific period (e.g. students) (Came and Tudor 2020). Additionally, they provide environments where students develop independence and learn life skills. HEIs are an important sector for investing in public health (Darker *et al*. 2023) and play a crucial role in leading and driving transformative changes in current and future societies, contributing to the well-being of people, places, and the planet (Dooris *et al*. 2021). Therefore, national and international initiatives should focus on positioning health promotion as a social and human development proposal over time (Lunnay *et al*. 2025).

### A salutogenic approach to health promotion

Within the context of recent approaches to the health promotion model, it is essential to address the salutogenic theoretical perspective (Antonovsky 1996). This approach emphasizes the factors responsible for well-being (Bhattacharya *et al*. 2020), focusing on identifying and leveraging internal or external personal resources to maintain health and psychological well-being (Mittelmark *et al*. 2022, Olsson *et al*. 2024). In contrast, the pathogenic model emphasizes illness, its treatment, and rehabilitation (Santos and Rodríguez 2023). In this manner, the salutogenic approach strengthens health promotion policies, prioritizing factors that positively affect health status, such as population resilience, social support, and coping strategies (Faria *et al*. 2024). The core concept of salutogenesis, the Sense of Coherence, refers to the extent to which individuals perceive life as comprehensible, manageable, and meaningful (Mohammed 2025). In higher education, this entails establishing coherent structures, providing accessible academic and psychosocial resources, and offering meaningful learning experiences while strengthening institutional health assets such as support networks, inclusive climates, and healthy settings (Brito 2018). Some studies have indicated that programmes with a salutogenic approach in higher education have the potential to improve health and quality of life, achieving notable results in the academic sphere (Morris-Paxton *et al*. 2019). However, operationalizing salutogenic theory at the university level also poses challenges (Faria *et al*. 2024).

Research on health promotion policies and initiatives in higher education has largely focused on the Global North (Dooris 2001a, 2001b, Soares *et al*. 2016, Newton *et al*. 2016, Sarmiento 2017, Brito 2018, Dooris *et al*. 2020, Hof-Nahor and Biswas 2020, Bachert *et al*. 2021, Garretsen *et al*. 2023, de Lima *et al*. 2025) and Oceania (Came and Tudor 2020, McDonald *et al*. 2021). This focus highlights a significant gap in documenting successful experiences of health-promoting universities in Latin America (Suárez-Reyes *et al*. 2021, Pérez-Wilson *et al*. 2022, Suárez-Reyes and Van Den Broucke 2023, Faria *et al*. 2024, Castillo-Rosales *et al*. 2025). Furthermore, there is a lack of documentation and significant gaps in knowledge regarding the implementation processes of practical experiences (Suárez-Reyes and Van Den Broucke 2016).

Therefore, this research presents the trajectory of a health promotion policy within a Latin American university and develops an analytical framework that strengthens the strategy and promotes the systematization of other health-promoting university experiences. It is essential to generate knowledge to understand the trajectories and dynamics of this type of initiative from a global perspective, thereby facilitating the replication of models and analytical frameworks in other contexts and addressing the growing challenges of fully integrating health promotion into university culture (Paduano *et al*. 2025).

This study evaluated the trajectory and level of consolidation of the health promotion policy at the Universidad Católica del Norte, Coquimbo (hereinafter, UCN), through a convergent mixed-methods design based on a framework of milestones (M), actors (A), processes (P), and challenges (C) (MAPC). The UCN has over 6000 students and 600 staff at its Coquimbo campus in northern Chile, offering a diverse range of undergraduate and postgraduate programmes in engineering, marine sciences, health, law, and education. The UCN is significant as it was the first higher education institution in Chile to declare its health promotion policy (Castillo-Rosales *et al*. 2025), and it is a key participant and leader in the Chilean Health Promoting Universities Network (Chilean HPU Network) as well as the Ibero-American Network of Health Promoting Universities (RIUPS). This study highlighted four key aspects: (i) identifying health promotion initiatives in university management processes as an institutional strategy (Dooris *et al*. 2021); (ii) bringing visibility to these types of initiatives, which are still scarce yet relevant in today’s world (Faria *et al*. 2024); (iii) contributing to the conceptual understanding of health promotion (Tafireyi and Grace 2024); and (iv) proposing a trajectory analysis method centred on the MAPC framework, which serves as a reference for the analysis of this type of initiative and facilitates synergism between national and international public health policies and higher education institutions.

## Materials and methods

### Study design

The research focused on a case study, which is an in-depth method for understanding complex issues within defined systems (Yin 2017). Specifically, the trajectory of the University Policy for the Promotion of Health and Quality of Life at the Universidad Católica del Norte (hereinafter, the VIVE UCN Policy) was analysed. The analysis was conducted from a longitudinal perspective (Neale 2020), concentrating on changes or consistencies in practices, perceptions, and interpretations over time (Hollstein 2021).

Using a phase and stage perspective (Saldaña 2003) and following the framework of milestones (M), actors (A), processes (P), and challenges (C), we structured the analysis of the case study. This MAPC framework is routinely employed by the Executive Committee of the VIVE UCN Policy for management and decision-making, enabling an examination of the present, past, and future aspects of a university health promotion policy (Castillo-Rosales *et al*. 2025). Specifically, the framework corresponds to the VIVE UCN Policy Evaluation Model (https://viveucn.ucn.cl/vive-ucn-coquimbo). Within this model, the analysis is structured around four analytical components: Milestones (M), defined as key events that have influenced the development of the VIVE UCN Policy; Actors (A), referring to people or groups belonging to the university who interact directly with the policy in activities or management; Processes (P), understood as the sequence of actions and institutional dynamics through which the Policy has evolved over time; and Challenges (C), which encompass the future tasks, management needs, and institutional adjustments required for the Policy’s consolidation, considering both internal perspectives and external obligations derived from national regulations and policy frameworks.

**Figure 1:**
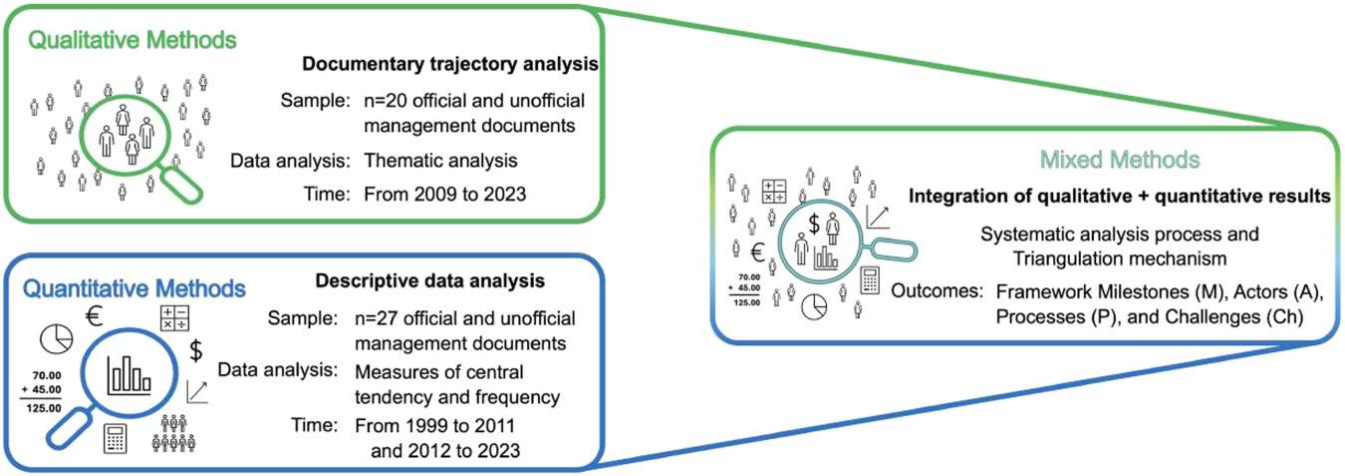
Graphical representation of the convergent mixed-methods design.

A mixed-methods design study that combines quantitative and qualitative research techniques was developed (Johnson and Onwuegbuzie 2004). Specifically a convergent design (QUAL + QUAN), with a focus on the fusion of information at the end. The integration of the methods was achieved through meta-inferences, which generated synergism among the methods (Younas *et al*. 2023). Thus, the convergent design has been employed to enhance the results and provide a deeper understanding of the policy (Creswell and Plano 2018) and educational issues (May 2025, Xu *et al*. 2025). Qualitative methodology was conducted through documentary analysis, following the approaches proposed by Morgan (2022) and Bowen (2009) (Qualitative phase section). In contrast, quantitative methods involved a descriptive statistical analysis of the documentary data to integrate the findings and achieve a comprehensive understanding of the study’s focus (Fig. 1).

### University health promotion policy VIVE UCN

The VIVE UCN Policy was created in a participatory manner, representing the entire university community: students, administrative staff, and academic staff. Its development featured a bottom-up approach, culminating in the institutional enactment of the university health promotion policy in 2012. To implement the VIVE UCN Policy, the health promotion university (HPU) initiative ‘*Encuéntrate UCN*’ serves as the primary platform for policy implementation. It consists of a weekly break (Wednesdays, 10:00–11:30), established by institutional decree, dedicated to the health and well-being of the university community through sports, cultural, and recreational activities, as well as health literacy initiatives. This initiative is highly valued by the university community (Castillo-Rosales *et al*. 2025). It is noted that some HPU initiatives were developed at different times from *Encuéntrate UCN*.

The objectives of the VIVE UCN Policy were to promote healthy settings and spaces for students, staff, and the broader university community, facilitate the development of professionals capable of responsibly managing their health and that of their setting, and enhance the quality of life by encouraging healthy lifestyles within the university community and their families. The HPU initiatives developed through ‘*Encuéntrate UCN*’ and in other university settings fell into the following categories: (i) healthy and sustainable lifestyles, (ii) physical activity, (iii) cultural participation, and (iv) community involvement.

### Data collection

Forty-seven digital documents (*n* = 47) were analysed over a time interval from 1999 to 2023. These documents included qualitative and quantitative information in varying volumes. For the qualitative analysis, the available records were the VIVE UCN Health Promotion and Quality of Life Policy (*n* = 1), the UCN Educational Project (*n* = 1), the decree of policy enactment (*n* = 1), the biannual evaluation reports of the VIVE UCN Policy (*n* = 5), internal management reports (*n* = 3), and documents related to the VIVE UCN Policy from national and international conferences (*n* = 9). For the quantitative analysis, the available records were annual management reports on the VIVE UCN Policy (*n* = 12), reports on student participation in general training courses and on university staff participation in personal improvement plans (*n* = 2), and internal annual management reports from UCN volunteers from 1999 to 2011 (*n* = 13). The conference documents and the internal annual management volunteer reports were analysed as unofficial documents. All documents analysed were prepared by UCN authorities and officials, except for the internal annual management volunteer reports, which were prepared by the students.

### Data analysis

#### Qualitative phase

A documentary analysis was conducted on official and unofficial records (*n* = 20) dated between 2009 and 2023 (Fig. 1). The analysis involved reviewing various information sources (Morgan 2022) and using a systematic procedure to select and examine their content, emphasizing ‘themes’ (Bowen 2009). For these analyses, the total number of documents was considered, with focus on segments containing texts referring to the VIVE UCN Policy. The qualitative information was selected, organized, and entered into the analysis software Atlas.ti 24. Using a flexible approach, a thematic analysis was initially performed to identify, analyse, and report patterns (themes) within the data (Braun and Clarke 2006, Humble and Mozelius 2022). Thus, a systematic deductive process was employed to identify milestones (M), actors (A), processes (P), and challenges (C), considering the policy’s chronology (time interval). Furthermore, an inductive analysis was conducted, uncovering new themes used to interpret and integrate the results.

Criteria for qualitative scientific rigour included transferability and credibility, achieved through the triangulation of data, methods, and results obtained during analysis meetings. Participants from the VIVE UCN Policy were incorporated into the formal analysis through researcher triangulation (Flick 2015). This procedure was divided into two phases: (i) the VIVE UCN Executive Committee in a formal meeting and (ii) researchers (i.e. authors and coauthors of the manuscript), who analysed the conclusions of the meeting held in Phase 1. Triangulation of information sources (Flick 2018) was achieved through the analysis of institutional and internal management reports on the VIVE UCN Policy, as well as reports from university and regional media outlets. Triangulation of methods (Denzin and Lincoln 2005) was also employed, with various information-gathering techniques used.

#### Quantitative phase

The total documents (*n* = 27), dated between 1999 and 2023 (Fig. 1), were processed and analysed in their numerical sections to generate systematized information regarding the participation of actors (A) in the different milestones (M) and processes (P) in the trajectory of the VIVE UCN Policy. The quantitative information consisted of selecting relevant data to specify, through descriptive statistical analysis (Holcomb 2016), measures of central tendency and frequency that reflect the level of involvement of university community members in each phase of the VIVE UCN Policy. The collected and analysed statistical data reflect the number of participations rather than the number of individuals involved in initiatives promoted by the VIVE UCN Policy. The data were used to create graphs and tables in Microsoft Excel.

Scientific rigour in the quantitative component was ensured through systematic data extraction, transparent coding procedures, and consistent application of frequency counts and measures of central tendency. These procedures enhanced internal validity, representational accuracy, and analytical coherence across the policy’s longitudinal trajectory.

### Ethical considerations

This study utilized unrestricted information for institutional-level analysis and may serve as a source of consultation. To protect confidentiality, sensitive details (e.g. names and positions) were omitted. This study adhered to the ethical guidelines of the American Psychological Association (APA): Beneficence and Non-Maleficence, Fidelity and Responsibility, Integrity, Justice, Respect for People’s Rights and Dignity (APA 2017).

## Results

The information interpretation and integration through the mixed path analysis of the HPU policy are organized according to the MAPC approach, focusing on the following subthemes: milestone in the trajectory of the VIVE UCN Policy (M), community university actors (A), the process of elaboration of the VIVE UCN Policy (P), and challenges of the policy of HPU (C).

### Milestones (M) in the trajectory of university health promotion policy

**Figure 2:**
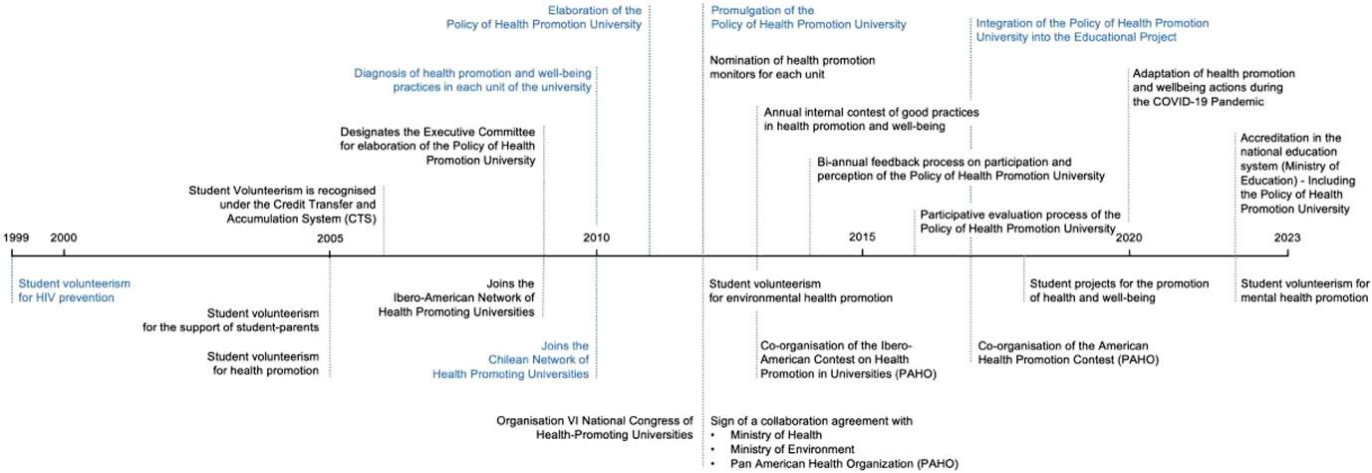
University health promotion policy timeline.

The various events that facilitated the implementation of the VIVE UCN Policy ranged from isolated initiatives voluntarily driven by students and university staff to those promoted institutionally or by university stakeholders. Figure 2 presents a timeline from 1999 to 2023, with institution-driven actions at the top and student-, university staff-, and stakeholder-driven actions at the bottom. The milestones that had the most significant impact on the policy’s trajectory are highlighted in blue (Fig. 2). Among the milestones were those in 1999, 2005, and 2013, which include student volunteer initiatives focused on sexual responsibility and HIV prevention, supporting student–parents, and promoting health and environmental well-being, marking the start of an active student movement role. By 2022, these efforts expanded to include mental health promotion (currently, all-volunteer groups are still active). The participation of UCN personnel in the IV International Congress of Health Promoting Universities in Spain triggered actions at the institutional and external levels. Thus, in 2009, the UCN designated the Executive Committee to develop the university health promotion policy and to adhere to the Ibero-American HPU Network, while in 2010, the institutional diagnosis of health promotion actions took place at the UCN, as did adherence to the Chilean HPU Network. In 2011, particular emphasis was placed on the participatory development of the VIVE UCN Policy, with students, administrative staff, and academic staff participating in policy design workshops. In 2012, the VIVE UCN Policy was formally institutionalized and enacted by the university authorities. These milestones illustrate the institution’s leading role in guiding the process and creating structured spaces for community participation. In 2017, a further milestone was the integration of the VIVE UCN Policy into the University’s Educational Project, itself developed through the participation of students, administrative staff, and academic staff. This incorporation positioned health promotion and well-being as a core pillar of comprehensive student education and led to the introduction of courses across all degree programmes that address competencies in physical activity promotion, mental health, social responsibility, and related themes.

### Community university actors (A) in the deployment of the policy of health promotion

The HPU initiatives implemented over time in the VIVE UCN Policy originated from various actors within the university community. Students and university staff acted as needs detectors and, working with the institution, promoted HPU initiatives. Because of their importance, specific initiatives at HPU became milestones (M) and processes (P) of the policy (e.g. health-promoting university week). Others, such as health promotion literacy activities (e.g. dialogues, healthy expos, and workshops) and the creation of healthy settings (e.g. hammocks and healthy challenges), aimed to address social determinants of health and, through a salutogenic approach, to promote health assets (e.g. ocean view and green areas). Internal university stakeholders used the ‘*Encuéntrate UCN*’ healthy break to conduct HPU initiatives in a meeting space that brought together various stakeholders. The ‘*Encuéntrate UCN*’ was one of the tools of the VIVE UCN Policy that documented many activities carried out during these hours (Table 1). Meanwhile, external stakeholders (e.g. the Ministry of Health and the Public Health System) promoted several HPU initiatives that focused on community engagement.

**Table 1:** HPU initiatives carried out in the framework of the VIVE UCN Policy.

Issue	Origin of the initiative	Funding	Activity
Healthy and sustainable lifestyles	Students	Volunteer work	Dialogue on mental health among university students^a^
		Internal fund	World No Tobacco Day
			Healthy challenges
			Preventive workshops on alcohol and drugs^a^
			Hammocks for rest and relaxation project Campus stairs with healthy messages
			Antistress campaign
	Students—VIVE UCN Policy	Internal fund	Academic stress management workshop^a^
	Students and staff	Internal fund	Mindfulness workshops
			Bach Flower workshop
			Restorative sleep workshop
			Self-care and well-being workshop
	Staff—VIVE UCN Policy	Internal fund	Healthy expo
			Health-promoting university week
	Staff	Internal fund	Education for a healthy old age
Physical activity	Staff	Internal fund	Family cycle ride
		Volunteer work	Bodybuilding workshops
	Staff—VIVE UCN Policy	Volunteer work	Running team training
		Internal fund	Family run
			Dance, Zumba, and rhythm workshops
Cultural participation	Students	Volunteer work	Photography workshop
	Staff	Internal fund	Cinematographic cycle
		Volunteer work	Forming of music bands
			Handmade textiles workshop
Community involvement	Students	Internal fund	Workshops on youth suicide prevention^a^
			International aids candlelight memorial
			Aquaponics project
			Cleaning and reforestation of the campus forest
			Ecological and composting day
			Gender and domestic violence workshops
			Workshops on breastfeeding
		Volunteer work	Education and parenting workshop
	Staff	Internal fund	Healing therapy with dogs
	University stakeholders	Public health system	Measles and mumps vaccination operation
			Blood donation campaign
			Preventive health assessments for adults

^a^Activities exclusively aimed at students’ participation.

Table 1 presents the funding sources required to support the various HPU initiatives, with ‘internal funds’ representing institutional funding, ‘volunteer work’ representing funding from student volunteers, and ‘public health system’ representing state funding. Table 1 shows that funds from the institution and voluntary contributions exceed those from stakeholders. The categories chosen to group the HPU initiatives include healthy and sustainable lifestyles, physical activity, cultural participation, and community involvement, aligning with the classification used in the institutional management of the VIVE UCN Policy. The activities shown in Table 1 were intended for all members of the university community, except those specified in the same table as being for students only.

**Figure 3:**
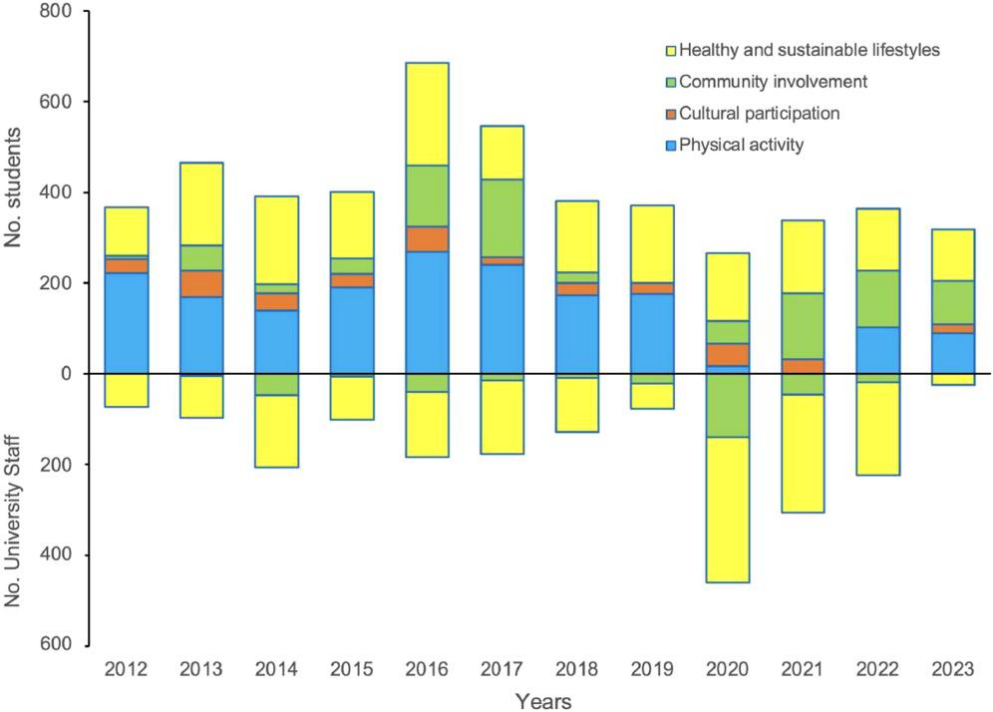
Participants: students (top) and university staff (bottom), differentiated by core thematic area from 2012 to 2023.

Figure 3 shows students’ and university staff’s responses to the HPU initiatives promoted under the VIVE UCN Policy framework. Participation data from 2012, when the policy was introduced, include activities proposed by the VIVE UCN executive committee and those started by students and university staff. Participation frequency is divided by year and distinguished between students (top) and university staff (bottom). An additional category highlights participants’ preferences for healthy and sustainable lifestyles, physical activity, cultural participation, and community involvement.

The HPU initiatives offered by the VIVE UCN Executive Committee were based on systematic preference surveys conducted within the university community. As a result, the participation of actors (Fig. 3) showed higher engagement among students than among university staff. Regarding preferred themes, initiatives focusing on healthy and sustainable lifestyles and physical activity were most popular among students, while community involvement and healthy and sustainable lifestyle activities were favoured by university staff. Following recognition of the VIVE UCN Policy in the 2017 educational project (Fig. 2), academic activities centred on health promotion and well-being were organized for students and awarded transferable credits. For university staff (excluding academics), the activities were included in annual job-training plans created by the institution and its employees, recognizing the hours spent on health promotion and wellness.

The design and organization of HPU initiatives stem from the implementation of the annual plan developed by the VIVE UCN Executive Committee, which incorporates activities proposed, designed, and delivered by members of the university community, such as student health volunteer groups. Regardless of their origin, all HPU initiatives are implemented within the framework of the VIVE UCN Policy, which provides the institutional structure for their delivery.

**Figure 4:**
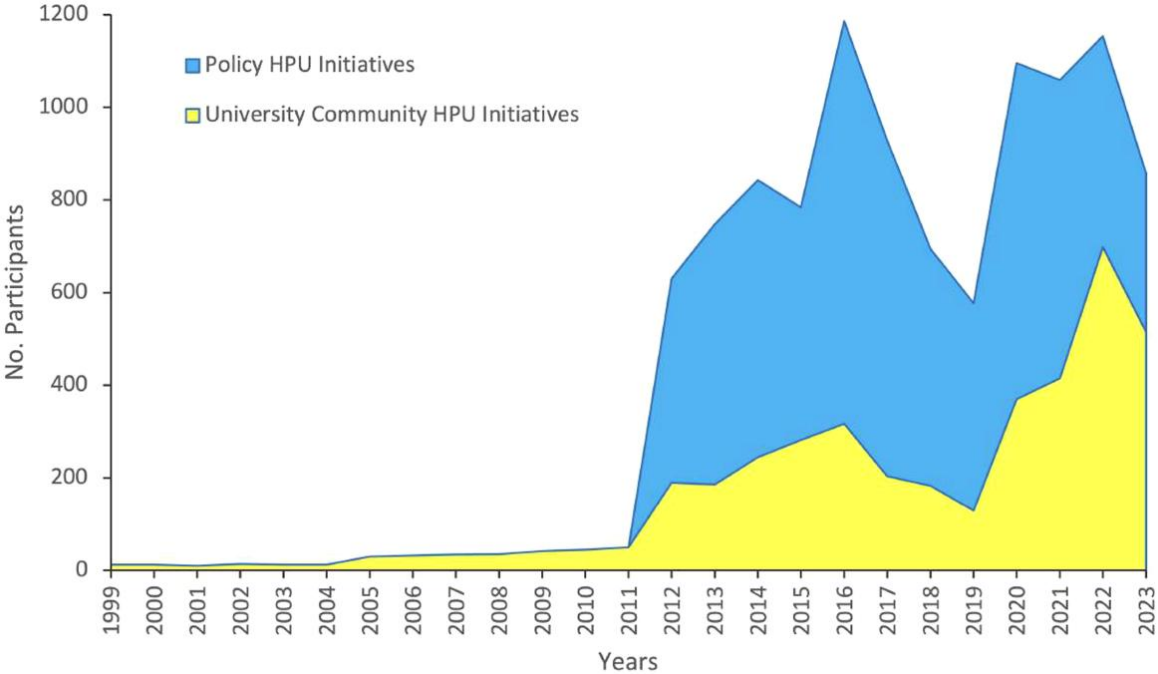
Participants in HPU initiatives, differentiated by activities of the VIVE UCN policy and others promoted by the university community.

When comparing participation in HPU initiatives (Fig. 4), those promoted through the annual activity plan designed by the VIVE UCN Executive Committee showed higher participation rates than those led and organized by university community members between 2012 and 2018. However, in subsequent years, participation tended to equalize across HPU initiatives of different origins. Notably, during the coronavirus disease 2019 (COVID-19) pandemic, the VIVE UCN Policy initiatives were carried out virtually and managed to restore previous participation levels. Before the VIVE UCN Policy was implemented, participation was consistently below 50, mainly from student volunteers.

### Process (P) of elaboration of the policy of HPU

From a process perspective, the VIVE UCN Policy is rooted in student initiatives (e.g. volunteering; Fig. 2) and then developed according to guidelines from university authorities who joined the Executive Committee established for the VIVE UCN Policy (Fig. 2), overseeing and guiding the development of the Policy of Health Promotion University. The VIVE UCN Executive Committee was formally appointed by institutional resolution of the university’s highest authority. It comprises representatives from academic affairs, administration and finance, communications, student services, and an expert in university health promotion, as well as student representatives (e.g. student leaders and health volunteer groups). Its composition remains unchanged over time; only the individuals who hold the positions it represents change.

**Figure 5:**
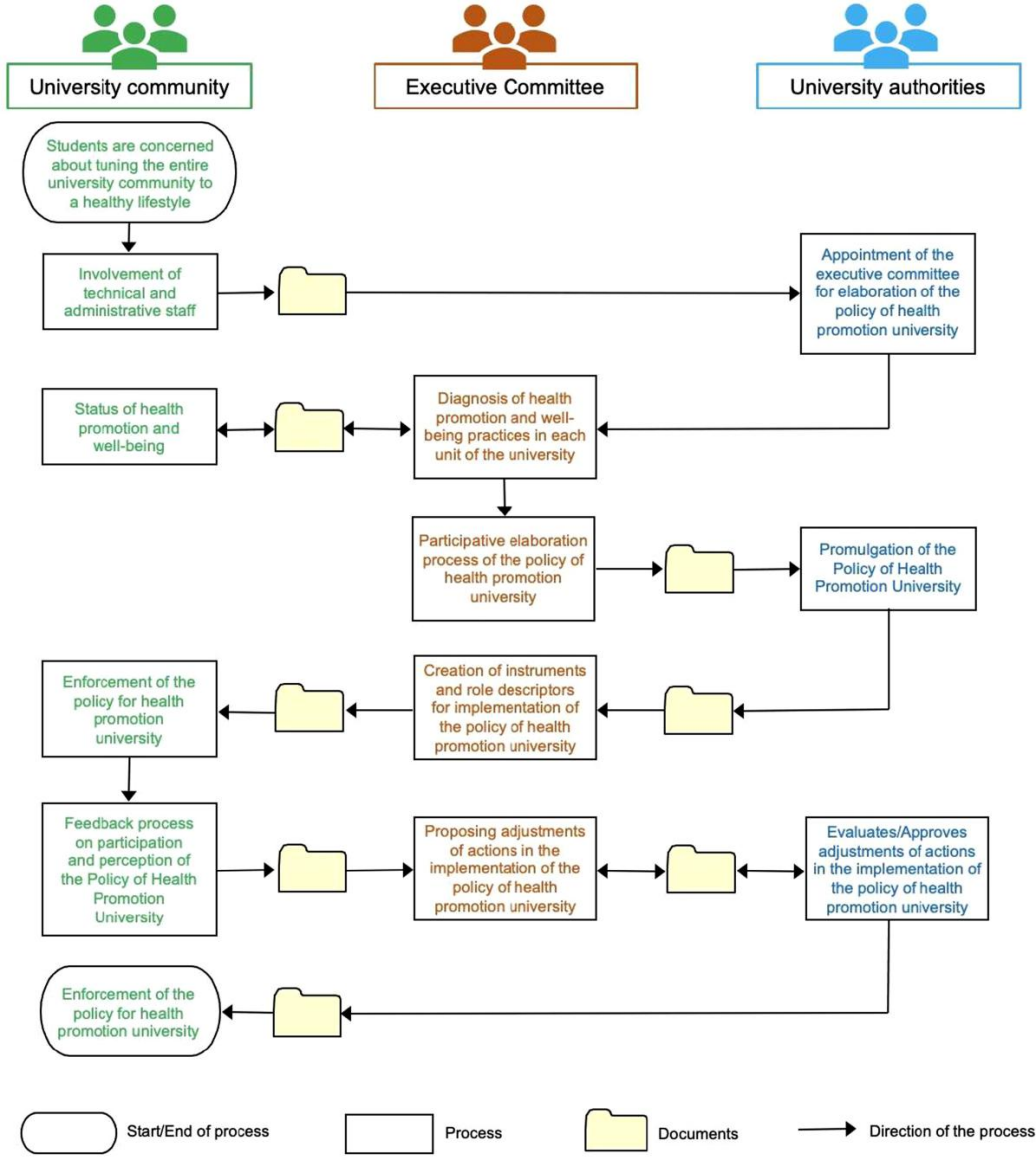
Elaboration process of the policy of the HPU.

The trajectory of the VIVE UCN Policy involves a series of actions that facilitated its implementation through coordination across various organizational and operational levels (Fig. 5). Therefore, creating the policy required different actors to perform specific tasks, fostering a collaborative cycle that embraced diverse visions and expectations. Figure 5 also shows the roles of the university community, the VIVE UCN Executive Committee, and the university authorities, highlighting the gradual involvement of each group and the actions they undertook, reflecting a systematic work process. Another key point is that the process started and ended within the university community.

### Challenges (C) of the policy of HPU: barriers and enablers

The enablers of VIVE UCN Policy include the creation of new healthy settings linked to the campus’s natural assets, the institutionalization of the weekly break (*Encuéntrate UCN*) as a structured and sustained health promotion space, and the participatory development of both the policy and its annual action plans.

Among the barriers identified are challenges related to leadership succession, stemming from student turnover due to graduation and the renewal of administrative staff and academic staff through retirement or new appointments. The difficulties in conveying the origins, rationale, and objectives of the VIVE UCN Policy stem from turnover. Second, lower levels of academic staff participation were noted.

## Discussion

Using an innovative, nascent methodology, this study analysed the trajectory of a university health promotion policy (VIVE UCN Policy) at a Chilean university. The framework, consisting of milestones (M), actors (A), processes (P), and challenges (C), facilitated highlighting key aspects of the policy and its evaluation and outlined common frameworks for implementing and evaluating such initiatives and policies in other contexts. The main findings include a chronology that ranges from voluntary, nonsystematic health promotion initiatives to the development of the VIVE UCN Policy and its incorporation into the institutional education project. The MAPC strategy also helped to unveil the intricate relationships within a university, stemming from the diversity of its internal and external actors, its management processes, or the constant challenges imposed by society in the areas of R&D and training professionals to become agents of change regarding social determinants (WHO 2021).

In terms of policy milestones (M), the numerous initiatives involving student participation stand out, such as volunteering for health issues and health promotion fairs (Table 1), which permeate the entire university community and lead to the VIVE UCN Policy milestones (Fig. 2). These milestones, promoted by students between 1999 and 2011, laid the foundations for the VIVE UCN Policy through a bottom-up process that contrasts with the usual top-down approaches (Darker *et al*. 2023). This social or community participation is considered crucial for implementing HPU initiatives, though it can be challenging to achieve (Griebler *et al*. 2017, Arroyo 2022). These opportunities provide knowledge and develop skills (Barranca-Enríquez *et al*. 2020), helping health promotion extend beyond the university’s boundaries. Furthermore, student volunteering has dual benefits, as it supports HPU initiatives and fosters new student skills and competencies (Suárez-Reyes *et al*. 2021). Another milestone recognized by the Chilean health system as an example of good practices in university health promotion is the institutional participation in the Chilean and Ibero-American HPU Networks, which has contributed to the continuous improvement of VIVE UCN Policy management. According to Darker *et al*. (2023), this involvement provides access to national and international reference frameworks and fosters institutional learning, benchmarking, and collaborative exchange with other universities committed to health promotion, as well as with the community (Buijs 2009). This analysis shows that integrating the VIVE UCN Policy into the institutional educational project in 2017 (Fig. 2) was a milestone that cemented health promotion within the institutional framework and served as a model for various university policies in Chile. This invites reflection on the implementation of these initiatives in educational contexts and emphasizes the importance of institutional political support in reinforcing them (Suárez-Reyes *et al*. 2021). Furthermore, the development of a sense of belonging (Castillo-Rosales *et al*. 2025) and the strengthening of interpersonal ties, both key elements of the salutogenic approach (Faria *et al*. 2024), reinforce empowerment and active community participation. This orientation supports shared responsibility, sustainability, and the transversal integration of health across university functions, fostering healthier and more meaningful academic environments (Brito 2018).

The active involvement of university community members and stakeholders (A) in developing and implementing the VIVE UCN Policy and various HPU initiatives helps outline thematic, temporal, and funding challenges. According to Fernandez *et al*. (2016), funding for the establishment of physical, social, and academic settings is crucial for promoting the mental well-being of students and university staff (Mather and Bam 2025). This supports the principle of community engagement within the 2019–30 health promotion strategy (PAHO 2022), the settings strategy (Suárez-Reyes *et al*. 2021), and the social determinants strategy (WHO 2021). In the present study, one finding emphasizes the active participation of students and university staff in HPU initiatives, their commitment to health promotion through various voluntary actions they have initiated, and their involvement in those promoted by the VIVE UCN Policy (Fig. 4; Table 1), thus visualizing the prominence of university staff who are often invisible in university management (Szekeres 2004). Another finding indicates that integrating health promotion into the University’s Educational Project increased student participation in VIVE UCN Policy initiatives (Fig. 3), aligning with the recommendations of Almomani *et al*. (2021), which aim to develop competencies and skills that enhance students’ health. Concerning the engagement of university staff in future studies, a distinction must be made between administrative staff and academic staff to clarify situations such as the high involvement of academic staff in planning university health promotion policies (Suárez-Reyes and Van Den Broucke 2023), contrasted with their low participation in HPU initiatives (Castillo-Rosales *et al*. 2025). This disparity may be explained by the complexity of higher education institutions as systems composed of multiple interacting components (Dooris *et al*. 2020), which enable actors to either disperse or concentrate their efforts according to personal priorities. Among academic staff, the perceived ‘value of time’ tends to be prioritized to meet demanding academic standards (Castillo-Rosales *et al*. 2025). Advancements in this area would enhance these actors’ structural and cultural commitments to health promotion (McDonald *et al*. 2021).

University authorities play a crucial role in implementing health promotion, both through their commitment to the strategy and the allocation of funding (Suárez-Reyes *et al*. 2021), while stakeholders (e.g. the Chilean Health System) strive to create a symbiotic relationship between the VIVE UCN Policy and their corporate initiatives’ interests. Another noteworthy aspect is that the families of university staff have also benefited from the policy, both directly through participation in activities, such as running events, and indirectly through the transfer of healthy practices and knowledge adopted by university staff within their households (Castillo-Rosales *et al*. 2025).

The creation of the VIVE UCN Executive Committee strengthened the process (P) of elaborating the VIVE UCN Policy. This collegial body (i.e. students, administrative staff, and academic staff), due to its experience in university management and health promotion, transitioned from an initiative desired by the university community to the establishment of a university health promotion policy through a bottom-up process (Fig. 5). Thus, these findings align with similar studies that highlight the role of strategic and effective leadership within these collegial bodies, which also provide institutional oversight during the implementation of these initiatives (Dooris *et al*. 2021, Suárez-Reyes *et al*. 2021), ensuring long-term institutional commitment that requires monitoring after implementation (Hay 2025). Although this case study shows evidence of university community participation in implementing the VIVE UCN Policy, progress must be made to actively involve students in decision-making bodies (e.g. the VIVE UCN Executive Committee) (Suárez-Reyes and Van Den Broucke 2023). Addressing this challenge will help overcome the structural difficulties universities face in changing, reorienting, and reconfiguring health promotion actions (e.g. Came and Tudor 2020). It is worth mentioning that the VIVE UCN Policy processes continue to this day, involving the transfer and feedback of information, knowledge, and dynamics (Fig. 5). This suggests that despite the challenge of having different individuals involved (i.e. the participants in 2012 are not the same as in 2023), the VIVE UCN Policy processes have permeated the university community and are connected to the development of community identity and belonging. However, the findings raise challenges regarding the importance of strengthening leadership succession (Yao *et al*. 2021) to ensure the sustainability of this policy initiative.

On reflection generated around the challenges (C), they highlight that, at the level of institutional policies, the VIVE UCN Policy should aim to highlight its role as a strategic instrument to reduce existing barriers, to give sustainability to this policy, and to consolidate a university setting that encourages and motivates healthier behaviours and decisions. In addition, it is necessary to increase the participation of academic staff through the implementation of research and teaching initiatives linked to health promotion (Ricci *et al*. 2022, Suárez-Reyes and Van Den Broucke 2023); promote inter- and intracommunity networking (Yeravdekar and Zodpey 2024); deepen health promotion structures, which are the basis for sustainable governance and development of these networks (Bachert *et al*. 2021); and prevent loss of consistency of institutional policies due to turnover of institutional actors (Bano *et al*. 2022). At the level of health promotion policy, the VIVE UCN Policy should promote intersectorality and social participation as a pillar of health promotion (PAHO 2022), highlight the salutogenic approach (Antonovsky 1996, García-Moya and Morgan 2016, Dooris *et al*. 2020), strengthen literacy processes to ensure the adoption of health promotion (Castillo-Rosales *et al*. 2025), follow group strategies to facilitate interventions (Souza *et al*. 2021), and consolidate the MAPC methodology from a conglomerate of past, present, and future perspectives for the projection of the VIVE UCN Policy. Finally, flexibility should be considered in university health promotion policies and HPU initiatives in the face of unplanned scenarios, such as the COVID-19 pandemic (Faria and Martínez-Riera 2023). At the research level, topics of interest include exploring the concept of salutogenesis in universities (Faria *et al*. 2024) and health promotion behaviours in members of the educational community (Dargahi *et al*. 2022) (e.g. García-Padilla *et al*. 2024, Fayda-Kinik and Kirisci-Sarikaya 2025), unravelling social–community links in health promotion (Arroyo 2022) by generating curricula on community health promotion problems, studying the link between health promotion and academic performance (Lee *et al*. 2022), and expanding into the relationship between health promotion and labour productivity (Aliberti *et al*. 2022). From a national perspective, the lessons learned during the process of institutionalizing this health promotion policy will help strengthen national policies (Thomas *et al*. 2025).

Regarding the limitations, at the design level, both qualitative and quantitative elements were equally weighted. The proposal could benefit from an explanatory sequential design (QUAN → QUAL) (e.g. Dias-Barbosa *et al*. 2023). This approach starts with quantitative data on the VIVE UCN Policy in the first phase, which will be complemented by qualitative techniques (i.e. interviews) in the second phase. On the other hand, the volume of available data prevented a more complex statistical analysis (e.g. relationships between variables), and the information gaps for specific periods (e.g. the pandemic), as well as the unavailability of information from UCN stakeholders, made it difficult to record data and evaluate the policy. This situation was remedied through a thorough analysis of the gathered information using a prior document processing procedure (Dulzaides Iglesias and Molina Gómez 2004), which enabled the detection of these information gaps. Specifically, there were no difficulties in accessing information as the documents were available to the VIVE UCN Executive Committee. Finally, regarding subjectivity and bias in qualitative research, this issue has been addressed in the literature (Zahle 2025); however, the use of different triangulation mechanisms (see Data analysis section) reduces the likelihood of bias. Furthermore, given its mixed-methods design, this approach compensates for the limitations of both qualitative and quantitative methods, creating an opportunity to address the research problem (Adu *et al*. 2022).

## Conclusion

This mixed-methods design study presents a framework focused on milestones (M), actors (A), processes (P), and challenges (C), enabling analysis and reflection on the trajectory of a health promotion policy within the educational community of a Chilean university. The MAPC approach also facilitates evaluating past and present stages and projecting desired future outcomes for the health promotion policy. Therefore, the MAPC analysis framework represents a methodological and conceptual advancement that enables the development of implementation and evaluation processes for managing institutional or health promotion policies in university settings and can be extrapolated to other case studies facing this gap.

The documentary analysis facilitated the identification of elements of interest in the implementation of the VIVE UCN Policy, highlighting its bottom-up gestation, from the initiative of university community members to the support of university authorities and the expertise in health promotion provided by the executive team in charge of the VIVE UCN Policy. These systematically executed instances enabled the transition from HPU initiatives to a health promotion policy within a university context. Thus, the different efforts channelled through institutionalized mechanisms enabled the progress and continuity of this policy. It is important to highlight that university community members, including student volunteers and staff, played a key role in implementing the VIVE UCN Policy and developing healthy settings. A salutogenic approach, which fostered a stronger sense of belonging and empowerment, contributed to creating healthy settings and improved the campus’s inherent health assets.

The implementation of an institutional health promotion policy, rather than isolated initiatives or short-term programmes, has ensured its permanence over time (13 years to date), as well as the allocation of resources and the enactment of official decrees that encourage and facilitate community participation. Another noteworthy finding was the inclusion of health promotion in the university’s educational project, which ensures its integration into educational trajectories by incorporating a salutogenic approach into courses and training plans for administrative staff. This resulted in strategies that strengthened the policy and advanced efforts to address the social determinants of health. Concerning the challenges of the VIVE UCN Policy in terms of its long-term projection, it must address the analysis and practical implications of its deployment, enhance its understanding among the university community, implement succession plans to ensure the continuity of its processes, and differentiate the participation of administrative staff from academic staff to focus on action plans.

Finally, the VIVE UCN Policy must be consistently articulated and updated in line with national public health policies and international guidelines. This will enable health promotion within the university context to create healthy settings. This will empower the university community members to act as agents of change and contribute to addressing the social determinants of health.

## Data Availability

This study utilized unrestricted information for analysis.
